# Environmental Induction of White–Opaque Switching in *Candida albicans*

**DOI:** 10.1371/journal.ppat.1000089

**Published:** 2008-06-13

**Authors:** Bernardo Ramírez-Zavala, Oliver Reuß, Yang-Nim Park, Knut Ohlsen, Joachim Morschhäuser

**Affiliations:** Institut für Molekulare Infektionsbiologie, Universität Würzburg, Würzburg, Germany; Johns Hopkins University School of Medicine, United States of America

## Abstract

*Candida albicans* strains that are homozygous at the mating type locus (*MTL* **a** or *MTL*α) can spontaneously switch at a low frequency from the normal yeast cell morphology (white) to an elongated cell type (opaque), which is the mating-competent form of the fungus. The ability to switch reversibly between these two cell types also contributes to the pathogenicity of *C. albicans*, as white and opaque cells are differently adapted to specific host niches. We found that in strain WO-1, a strain in which genomic alterations have occurred, but not in other tested strains, switching from the white to the opaque phase can also be induced by environmental conditions. Transient incubation of white cells under anaerobic conditions programmed the cells to switch *en masse* to the opaque phase. The anaerobic induction of white–opaque switching was controlled by the transcription factor *CZF1*, which in heterozygous *MTL* **a**/α cells regulates filamentous growth under embedded, hypoxic conditions. Intriguingly, passage of white cells of strain WO-1 through the mouse intestine, a host niche in which the cells are likely to be exposed to anaerobic conditions, resulted in a strongly increased frequency of switching to the opaque phase. These results demonstrate that white–opaque switching is not only a spontaneous process but, in combination with genomic alterations, can also be induced by environmental signals, suggesting that switching and mating of *C. albicans* may occur with high efficiency in appropriate niches within its human host.

## Author Summary

Some strains of the opportunistic fungal pathogen *Candida albicans* can reversibly switch from the normal yeast morphology (white) to an elongated cell type termed opaque, which is the mating-competent form of *C. albicans*. *C. albicans* strains first have to become homozygous at the mating type locus to be able to switch to the usually less virulent opaque phase. As white–opaque switching occurs spontaneously only at a low frequency, the encounter of opaque cells of different strains and mating types is expected to be an extremely rare event and little recombination between strains occurs in nature. We found that genomic alterations may render white–opaque switching environmentally inducible in certain *C. albicans* strains. A transient incubation of such a strain under anaerobic conditions induced mass switching of white cells to the opaque phase, and this induction was mediated by the transcription factor Czf1p. Most strikingly, passage of white cells through the mammalian intestine, an environment in which the cells encounter anaerobic conditions, also stimulated switching to the opaque phase. Therefore, some strains of *C. albicans* may be induced to switch to the mating-competent opaque phase in response to environmental signals, giving them an opportunity for efficient genetic exchange in appropriate host niches.

## Introduction

The yeast *Candida albicans* is a member of the microbial flora of the gastrointestinal and urogenital tract in many healthy people, but it can also cause serious infections when host defenses are compromised. In severely immunosuppressed patients, *C. albicans* can disseminate and infect virtually all body locations, indicating that *C. albicans* is able to adapt to many different environmental conditions within its host. The great morphological variability of *C. albicans* contributes to its capacity to spread to and establish itself within new host niches [1]. In response to environmental cues, e.g. the presence of serum, an increase in pH and temperature, or starvation conditions, *C. albicans* transitions from the budding yeast form to filamentous growth forms (true hyphae and pseudohyphae). This yeast-hyphal dimorphism, which includes dramatic alterations in the gene expression pattern, is important for the pathogenicity of *C. albicans* and mutants that are locked in the yeast or the filamentous form are attenuated for virulence [2],[3].

In addition to the yeast-hyphal transition, *C. albicans* can also switch from the normal, round-to-oval yeast cell morphology (white) to an elongated cell type with an altered surface structure, which has been termed opaque because of the appearance of the colonies produced by these cells on agar plates [4]. It was recently discovered that opaque cells are the mating-competent form of *C. albicans* and only strains that are homozygous at the mating type locus (*MTL* **a** or *MTL*α) can switch from the white to the opaque form and mate with opaque cells of the opposite mating type [5],[6]. The majority of *C. albicans* strains are *MTL* **a**/α heterozygous and produce the **a**1–α2 repressor, which is encoded by the two mating type loci and inhibits white-opaque switching. However, these strains can become *MTL* homozygous and switching-competent by loss of one homologue of the *MTL* carrying chromosome 5 and duplication of the remaining homologue or by mitotic recombination [7].

White-opaque switching also contributes to a better adaptation of *C. albicans* to new host niches. While white cells are much more virulent than opaque cells after intravenous infection, opaque cells are better able than white cells to infect skin [8],[9]. Switching between the white and opaque phases occurs spontaneously at a relatively low frequency, such that white or opaque cell populations usually contain about 0.1% of cells of the opposite phase. This frequency of switching allows a semi-stable maintenance of the white and opaque phases once they are established and at the same time ensures that some cells in the population are preadapted to altered environmental conditions upon encountering new host niches [10]. Since opaque cells are unstable at 37°C, it is believed that switching from the white to the opaque form may be relevant especially in host niches with lower temperatures, like skin, which also facilitates mating [11].

The molecular basis of the control of white-opaque switching has recently been elucidated [12]–[14]. In *MTL* heterozygous strains, the **a**1–α2 repressor inhibits expression of the *WOR1* gene, which encodes a transcription factor that induces switching of white cells to the opaque phase and also upregulates its own expression. White cells of *MTL* homozygous strains express *WOR1* at very low levels, but stochastic expression of *WOR1* above a threshold level in some cells in a population results in a positive feedback loop that generates the high Wor1p levels required to switch to and maintain the opaque phase. Similarly, a disturbance in *WOR1* expression in some cells of an opaque cell population may decrease Wor1p levels below the necessary threshold and result in switching to the white phase. These results beautifully explain the stochastic nature of white-opaque switching.

Here we report that white-opaque switching does not only occur spontaneously at low frequency but can also be induced by environmental signals. We found that a transient incubation of white cells under anaerobic conditions resulted in mass switching to the opaque phase, which was mediated by the transcription factor Czf1p and depended on additional genomic alterations. As the anaerobically induced white-opaque switching also occurred at 37°C and in the mammalian gastrointestinal tract, these findings have major implications for our understanding of the control of the *C. albicans* life cycle by the environmental conditions encountered within its host.

## Results

### Anaerobic conditions induce white-opaque switching in strain WO-1

**Figure 1:**
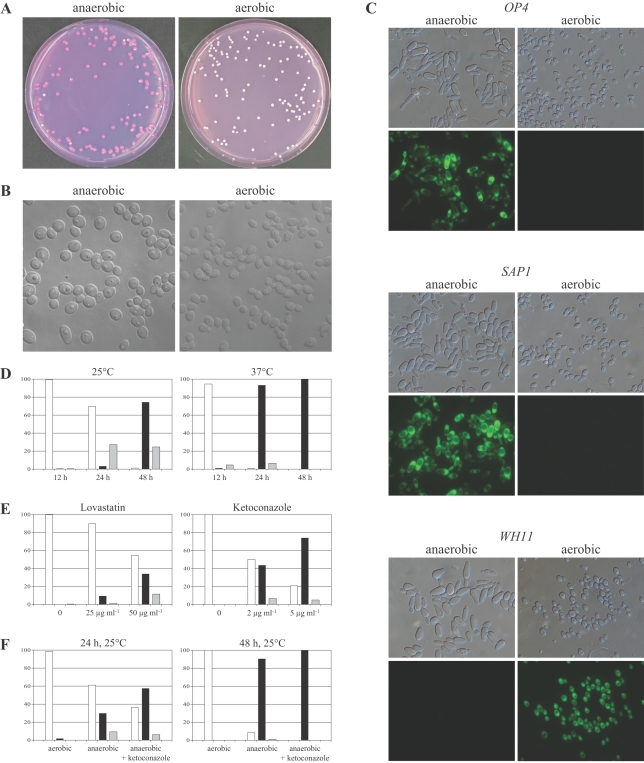
Anaerobic conditions induce white-opaque switching in *C. albicans* strain WO-1. (A) White-phase cells were spread on Lee's agar plates with phloxine B, incubated for 2 days at 25°C under anaerobic conditions, and then grown for 7 days under aerobic conditions (left) or grown for 7 days under aerobic conditions without a previous anaerobic incubation (right). (B) Microscopic appearance of white-phase cells that had been incubated for 2 days at 25°C on Lee's agar plates under anaerobic (left) or aerobic conditions (right). Cells in both panels are shown at the same magnification. (C) Expression of the opaque-phase-specific *OP4* and *SAP1* genes and the white-phase-specific *WH11* gene after anaerobic induction of white-opaque switching. Derivatives of strain WO-1 expressing the *GFP* reporter gene under control of the *OP4*, *SAP1*, or *WH11* promoter were incubated under anaerobic (left) or aerobic (right panels) conditions as described in (A). Cells from the resulting opaque colonies after the anaerobic incubation and from white colonies on the control plates were grown to log phase in liquid Lee's medium at 25°C and observed by microscopy. Shown are corresponding differential interference contrast (top panels) and epifluorescence micrographs (bottom panels) of the cells. (D) Percentage of white (white bars), opaque (black bars), and mixed white/opaque colonies (grey bars) after incubation of white-phase cells on Lee's agar plates for 12 h, 24 h, and 48 h under anaerobic conditions at 25°C (left) or 37°C (right). (E) Percentage of white, opaque, and mixed white/opaque colonies after incubation of white-phase cells for 48 h in liquid Lee's medium containing the indicated amounts of lovastatin or ketoconazole and subsequent growth of the cells on Lee's agar plates. (F) Percentage of white, opaque, and mixed white/opaque colonies after incubation of white-phase cells on agar plates with or without 5 µg ml^−1^ ketoconazole for 24 h or 48 h at 25°C under anaerobic conditions. Control plates were incubated under aerobic conditions.

In experiments aimed at elucidating the function of phase-specific genes we incubated strain WO-1, the *MTL*α strain in which white-opaque switching was originally discovered, and mutants derived from it on Lee's agar plates at 25°C under anaerobic conditions (see Materials and Methods). Under these conditions the cells soon ceased to grow and formed only microcolonies consisting of few cells. When the plates were then transferred to aerobic conditions to allow growth and formation of visible colonies, we made the striking observation that virtually all white cells of strain WO-1 had switched to the opaque phase whereas they remained in the white phase when the plates were kept under aerobic conditions without the transient anaerobic incubation (Fig. 1A). Microscopic examination of the cells recovered from the plates directly after the anaerobic incubation showed that they did not exhibit the elongated opaque morphology but formed enlarged round cells (Fig. 1B). However, when these cells were plated on Lee's agar plates and incubated at room temperature under aerobic conditions, most of them produced opaque colonies consisting of cells with the typical opaque morphology. The opaque cells expressed the opaque-phase-specific *OP4* and *SAP1* genes and had downregulated expression of the white-phase-specific *WH11* gene (Fig. 1C, left panels). In contrast, cells that were kept under aerobic conditions exhibited the white cell morphology and did not detectably express the *OP4* and *SAP1* genes, but continued to express the *WH11* gene (Fig. 1C, right panels). These results indicated that a transient incubation under anaerobic conditions programs white cells to switch to the opaque phase. In control experiments we tested if the heterozygous *MTL* **a**/α strain SC5314 would also switch to the opaque phase when incubated under anaerobic conditions. However, no opaque colonies were produced by this strain, suggesting that, as for spontaneous white-opaque switching, *MTL* homozygosity is a prerequisite for the anaerobically induced switching of white cells to the opaque phase.

Anaerobic conditions are likely to be encountered in host niches like the intestinal tract, i.e., at the body temperature of 37°C at which opaque cells are unstable. We therefore tested whether anaerobic conditions would also induce white-opaque switching at 37°C. Unexpectedly, switching of white cells to the opaque phase was even accelerated at 37°C. While at 25°C an anaerobic incubation for 2 days was required to induce most cells to switch to the opaque phase, nearly complete switching was observed already after 24 h of incubation under anaerobic conditions at 37°C. Shorter anaerobic incubation times resulted in little (at 37°C) or no significant (at 25°C) switching (Fig. 1D). The more efficient switching at 37°C presumably does not reflect a specific temperature signal but may rather be due to the increased metabolic activity at the higher temperature.

As the cells could not grow on Lee's agar plates under anaerobic conditions, we considered the possibility that switching to the opaque phase might have been induced by growth inhibition and not by the anaerobic conditions *per se*. Therefore, we also incubated white cells in distilled water for 48 h and then plated them on Lee's medium for colony formation. Under these conditions the cells remained in the white phase and showed only the normal spontaneous switching frequency. As an alternative means to generate anaerobic conditions, we cultured the cells in liquid Lee's medium treated for 10 min with nitrogen gas to remove the oxygen. After 2 days of incubation at 37°C, about 9% of the cells formed opaque colonies or mixed white/opaque colonies, whereas no opaque colonies were observed when the cells were incubated in the same way in medium not treated with nitrogen. While switching was not as efficiently induced under these conditions as during incubation on solid medium in an anaerobic jar, presumably because treatment of the liquid medium with nitrogen gas did not generate completely anaerobic conditions, these experiments nevertheless confirmed that anaerobic or hypoxic conditions induced switching of strain WO-1 from the white to the opaque phase.

Anaerobic conditions result in ergosterol depletion and accumulation of precursor molecules, as several enzymes in the ergosterol biosynthesis pathway require oxygen for their activity, and yeast cells may sense anaerobic conditions by measuring the levels of sterols and biosynthetic intermediates [15],[16]. To investigate whether, similar to anaerobic conditions, inhibition of ergosterol biosynthesis would also induce white-opaque switching, white cells of strain WO-1 were incubated for 2 days at 30°C in Lee's medium containing lovastatin or ketoconazole. As can be seen in Fig. 1E, treatment with these ergosterol biosynthesis inhibitors induced white-opaque switching in a dose-dependent manner, with ketoconazole exhibiting the strongest effect (about 80% opaque cells at 5 µg ml^−1^ ketoconazole). The presence of ketoconazole also accelerated the anaerobically induced switching when the cells were incubated on agar plates at 25°C (Fig. 1F). These experiments supported the hypothesis that anaerobic conditions induce white-opaque switching by causing the depletion of ergosterol and/or the accumulation of sterol biosynthesis intermediates in the cells.

### The transcription factor *CZF1* controls anaerobically induced white-opaque switching

The finding that white cells of strain WO-1 can be efficiently induced to switch to the opaque phase by anaerobic conditions suggested that transcription factors that regulate aerobic/anaerobic gene expression might also be involved in the control of white-opaque switching. We therefore tested if expression of such transcription factors from an inducible promoter would cause white cells of strain WO-1 to switch to the opaque phase. Several candidate transcription factors were chosen for this analysis. *UPC2* encodes a transcription factor that is required for sterol uptake and for the upregulation of *ERG* genes under anaerobic/hypoxic conditions and in the presence of sterol biosynthesis inhibitors [17],[18]. The *HAP41*, *HAP42* and *HAP43* genes are the *C. albicans* homologues of *S. cerevisiae HAP4*, which encodes a subunit of the Hap2/3/4/5p complex involved in aerobic gene regulation and whose expression levels control the activity of this transcription factor [19],[20]. In *C. albicans*, the Hap2/3/4/5p complex is also involved in the regulation of filamentous growth [20]. *RFG1* is the *C. albicans* homologue of *S. cerevisiae ROX1*, which is a transcription factor that is itself regulated by Hap1p to mediate repression of hypoxic genes. Although *RFG1* is not involved in the regulation of aerobic/anaerobic gene expression in *C. albicans*, it is an important regulator of filamentous growth in *MTL* **a**/α cells [21],[22]. Similarly, *CZF1* encodes a transcription factor that is required for filamentous growth of *C. albicans* under embedded/hypoxic conditions [23], and we hypothesized that it might also be involved in the regulation of another morphogenetic program, white-opaque switching, in *MTL* homozygous strains under anaerobic conditions. The coding regions of these transcription factors were placed under the control of a tetracycline-inducible promoter [24] and the expression cassettes integrated into the genome of strain WO-1. A control construct contained the *GFP* reporter gene instead of a transcription factor. Two independent transformants containing a single copy of the inducible expression cassette were kept in each case (see Table 1 for a description of all strains used in this study). White cells of the parental strain WO-1 and its derivatives were grown for 24 h at room temperature in Lee's medium in the presence or absence of 50 µg ml^−1^ doxycycline and then plated on Lee's agar plates to determine the percentage of white and opaque colonies. In the absence of doxycycline all strains remained in the white phase and showed only the normal, low-frequency switching to the opaque phase. In contrast, doxycycline-induced expression of the *CZF1* gene resulted in almost complete switching of the cells to the opaque phase, whereas expression of the other genes had no effect (Table 2). These results implicated *CZF1* in the regulation of white-opaque switching, a finding that has recently been reported independently by two other groups [25],[26].

**Table 1:** *C. albicans* strains used in this study.

Strain	Parent	Relevant genotype or characteristics	Reference
Wild-type strains			
WO-1		*MTL*α	[4]
12C		*MTL* **a**	[47]
19F		*MTL*α	[47]
L26		*MTL* **a**	[6]
P37005		*MTL* **a**	[6]
P57072		*MTL*α	[48]
P78048		*MTL*α	[48]
Reporter strains expressing *GFP* from phase-specific promoters			
WUGO4A and -B	WO-1	*ura3*Δ::*FRT/ura3*Δ::*FRT*	[49]
		*OP4*/*op4*::P*_OP4_-GFP-URA3*	
WUGS1A and -B	WO-1	*ura3*Δ::*FRT/ura3*Δ::*FRT*	[50]
		*SAP1*/*sap1*::P*_SAP1_-GFP-URA3*	
WUGW11A and -B	WO-1	*ura3*Δ::*FRT/ura3*Δ::*FRT*	[50]
		*WH11*/*wh11*::P*_WH11_-GFP-URA3*	
*czf1*Δ mutants			
WCZF1M1A and -B	WO-1	*CZF1*/*czf1*Δ::*SAT1-FLIP* a	this study
WCZF1M2A	WCZF1M1A	*CZF1*/*czf1*Δ::*FRT*	this study
WCZF1M2B	WCZF1M1B	*CZF1*/*czf1*Δ::*FRT*	this study
WCZF1M3A	WCZF1M2A	*czf1*Δ::*FRT*/*czf1*Δ::*SAT1-FLIP*	this study
WCZF1M3B	WCZF1M2B	*czf1*Δ::*FRT*/*czf1*Δ::*SAT1-FLIP*	this study
WCZF1M4A	WCZF1M3A	*czf1*Δ::*FRT*/*czf1*Δ::*FRT*	this study
WCZF1M4B	WCZF1M3B	*czf1*Δ::*FRT*/*czf1*Δ::*FRT*	this study
WCZF1MK1A	WCZF1M4A	*czf1*Δ::*FRT*/*CZF1-SAT1-FLIP*	this study
WCZF1MK1B	WCZF1M4B	*czf1*Δ::*FRT*/*CZF1-SAT1-FLIP*	this study
WCZF1MK2A	WCZF1MK1A	*czf1*Δ::*FRT*/*CZF1-FRT*	this study
WCZF1MK2B	WCZF1MK1B	*czf1*Δ::*FRT*/*CZF1-FRT*	this study
*wor1*Δ mutants			
WWOR1M1A and -B	WO-1	*WOR1*/*WOR1*/*wor1*Δ::*SAT1-FLIP*	this study
WWOR1M2A	WWOR1M1A	*WOR1*/*WOR1*/*wor1*Δ::*FRT*	this study
WWOR1M2B	WWOR1M1B	*WOR1*/*WOR1*/*wor1*Δ::*FRT*	this study
WWOR1M3A	WWOR1M2A	*WOR1*/*wor1*Δ::*FRT*/*wor1*Δ::*SAT1-FLIP*	this study
WWOR1M3B	WWOR1M2B	*WOR1*/*wor1*Δ::*FRT*/*wor1*Δ::*SAT1-FLIP*	this study
WWOR1M4A	WWOR1M3A	*WOR1*/*wor1*Δ::*FRT*/*wor1*Δ::*FRT*	this study
WWOR1M4B	WWOR1M3B	*WOR1*/*wor1*Δ::*FRT*/*wor1*Δ::*FRT*	this study
WWOR1M5A	WWOR1M4A	*wor1*Δ::*FRT*/*wor1*Δ::*FRT*/*wor1*Δ::*SAT1-FLIP*	this study
WWOR1M5B	WWOR1M4A	*wor1*Δ::*FRT*/*wor1*Δ::*FRT*/*wor1*Δ::*SAT1-FLIP*	this study
WWOR1M6A	WWOR1M5A	*wor1*Δ::*FRT*/*wor1*Δ::*FRT*/*wor1*Δ::*FRT*	this study
WWOR1M6B	WWOR1M5B	*wor1*Δ::*FRT*/*wor1*Δ::*FRT*/*wor1*Δ::*FRT*	this study
Strains expressing transcription factors or *GFP* from the Tet-inducible promoter			
WTET1-CZF1A and -B	WO-1	*ADH1*/*adh1*::P_tet_-*CZF1*	this study
WTET1-HAP41A and -B	WO-1	*ADH1*/*adh1*::P_tet_-*HAP41*	this study
WTET6-HAP42A and -B	WO-1	*ADH1*/*adh1*::P_tet_-*HAP42*	this study
WTET1-HAP43A and -B	WO-1	*ADH1*/*adh1*::P_tet_-*HAP43*	this study
WTET6-RFG1A and -B	WO-1	*ADH1*/*adh1*::P_tet_-*RFG1*	this study
WTET6-UPC2A and -B	WO-1	*ADH1*/*adh1*::P_tet_-*UPC2*	this study
WTET6-WOR1A and -B	WO-1	*ADH1*/*adh1*::P_tet_-*WOR1*	this study
WNIM1A and -B	WO-1	*ADH1*/*adh1*::P_tet_-*GFP*	this study
WCZF1M4TET1-CZF1A	WCZF1M4A	*czf1*Δ::*FRT*/*czf1*Δ::*FRT*	this study
		*ADH1*/*adh1*::P_tet_-*CZF1*	
WCZF1M4TET1-CZF1B	WCZF1M4B	*czf1*Δ::*FRT*/*czf1*Δ::*FRT*	this study
		*ADH1*/*adh1*::P_tet_-*CZF1*	
WCZF1M4TET6-WOR1A	WCZF1M4A	*czf1*Δ::*FRT*/*czf1*Δ::*FRT*	this study
		*ADH1*/*adh1*::P_tet_-*WOR1*	
WCZF1M4TET6-WOR1B	WCZF1M4B	*czf1*Δ::*FRT*/*czf1*Δ::*FRT*	this study
		*ADH1*/*adh1*::P_tet_-*WOR1*	
WWOR1M6TET1-CZF1A	WWOR1M6A	*wor1*Δ::*FRT*/*wor1*Δ::*FRT*/*wor1*Δ::*FRT*	this study
		*ADH1*/*adh1*::P_tet_-*CZF1*	
WWOR1M6TET1-CZF1B	WWOR1M6B	*wor1*Δ::*FRT*/*wor1*Δ::*FRT*/*wor1*Δ::*FRT*	this study
		*ADH1*/*adh1*::P_tet_-*CZF1*	
WWOR1M6TET6-WOR1A	WWOR1M6A	*wor1*Δ::*FRT*/*wor1*Δ::*FRT*/*wor1*Δ::*FRT*	this study
		*ADH1*/*adh1*::P_tet_-*WOR1*	
WWOR1M6TET6-WOR1B	WWOR1M6B	*wor1*Δ::*FRT*/*wor1*Δ::*FRT*/*wor1*Δ::*FRT*	this study
		*ADH1*/*adh1*::P_tet_-*WOR1*	
Strains carrying an ectopically integrated additional *WOR1* copy			
19F-WOR1	19F	*MTL*α	this study
		*ACT1*/*act1*::*WOR1-caSAT1*	
L26-WOR1A and -B	L26	*MTL* **a**	this study
		*ACT1*/*act1*::*WOR1-caSAT1*	
P37005-WOR1A and -B	P37005	*MTL* **a**	this study
		*ACT1*/*act1*::*WOR1-caSAT1*	
P78048-WOR1A and -B	P78048	*MTL*α	this study
		*ACT1*/*act1*::*WOR1-caSAT1*	
Additional stocks of WO-1 and mutant derivatives			
Soll 1		*WOR1*/*WOR1*/*WOR1*	T. Srikantha
Soll 1 M1	Soll 1	*WOR1*/*WOR1*/*wor1*Δ::*SAT1-FLIP*	this study
Broad		*WOR1*/*WOR1*/*WOR1*	J. Berman
Broad M1	Broad	*WOR1*/*WOR1*/*wor1*Δ::*SAT1-FLIP*	this study
Soll2		*WOR1*/*WOR1*	J. Berman
Soll 2 M1	Soll 2	*WOR1*/*wor1*Δ::*SAT1-FLIP*	this study
WO-1mut	Soll 1	*WOR1*/*WOR1*/*WOR1*	this study
WO-1mut M1	WO-1mut	*WOR1*/*WOR1*/*wor1*Δ::*SAT1-FLIP*	this study

a *SAT1-FLIP* denotes the *SAT1* flipper cassette.

**Table 2:** Frequency of white-opaque switching in strains expressing transcription factors under control of a Tet-inducible promoter.

Strain	Gene expressed from P_tet_	no. of white, opaque, and mixed white/opaque (w/o) colonies^a^					
		+Dox			−Dox		
		white	opaque	w/o	white	opaque	w/o
WO-1	-	6,185	2	7	8,650	0	12
WNIM1A	*GFP*	6,170	3	10	6,990	3	14
WNIM1B	*GFP*	6,875	3	6	6,145	1	10
WTET1-UPC2A	*UPC2*	9,235	4	19	15,533	2	15
WTET1-UPC2B	*UPC2*	11,165	2	31	13,675	3	33
WTET1-HAP41A	*HAP41*	12,570	4	31	14,535	5	64
WTET1-HAP41B	*HAP41*	9120	11	20	12,410	9	50
WTET1-HAP42A	*HAP42*	12,070	3	29	13,124	4	44
WTET1-HAP42B	*HAP42*	13,400	3	40	13,505	2	47
WTET1-HAP43A	*HAP43*	6,430	5	89	11,220	8	162
WTET1-HAP43B	*HAP43*	7,175	2	49	10,565	6	42
WTET1-RFG1A	*RFG1*	12,030	2	43	14,505	9	73
WTET1-RFG1B	*RFG1*	10,290	1	20	10,160	4	47
WTET1-CZF1A	*CZF1*	120	6,435	23	8,055	4	50
WTET1-CZF1B	*CZF1*	96	4,825	5	8,100	3	29

a Results are from 4–5 independent experiments performed on separate days.

**Figure 2:**
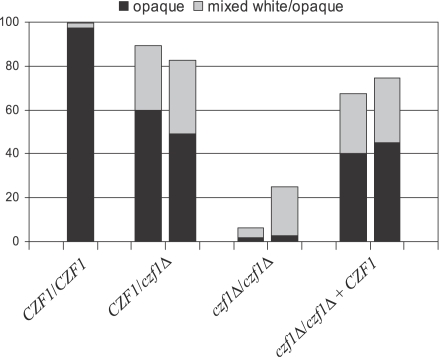
The transcription factor Czf1p is required for anaerobically induced white-opaque switching. White-phase cells of the wild-type strain WO-1 (*CZF1*/*CZF1*) and two independently constructed heterozygous (*CZF1*/*czf1*Δ) and homozygous (*czf1*Δ/*czf1*Δ) *czf1* mutants and complemented strains (*czf1*Δ/*czf1*Δ+*CZF1*) were incubated for 2 days under anaerobic conditions at 25°C on Lee's agar plates and subsequently grown for one week under aerobic conditions to determine the percentage of opaque (black bars) and mixed white/opaque colonies (grey bars).

To determine whether *CZF1* is also required for anaerobically induced white-opaque switching, we deleted the gene from strain WO-1. Two independent series of heterozygous and homozygous *czf1*Δ mutants as well as complemented strains in which a functional *CZF1* copy was reintegrated were constructed using the *SAT1*-flipping strategy [27]. Deletion of *CZF1* did not abolish white-opaque switching, as all strains could spontaneously switch to the opaque phase (data not shown). However, the anaerobically induced switching of white cells to the opaque phase was drastically decreased, albeit not eliminated, in mutants lacking *CZF1*, and the heterozygous and complemented strains also exhibited a slightly reduced efficiency of induced switching (Fig. 2). These results demonstrated that *CZF1* plays an important role in the anaerobic induction of white-opaque switching.

**Figure 3:**
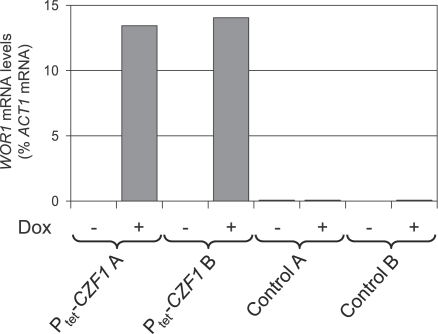
Tetracycline-induced *CZF1* expression results in *WOR1* induction. Strains expressing *CZF1* (P_tet_-*CZF1*) or *GFP* (control) from the Tet-inducible promoter were grown for 18 h at 30°C in liquid Lee's medium with (+) or without (−) 50 µg ml^−1^ doxycycline (Dox). RNA was isolated from the cultures and *WOR1* transcript levels were quantified by real-time RT-PCR and normalized to *ACT1* transcript levels. Two independently constructed strains (A and B) were used in each case.

*WOR1* encodes the master regulator of white-opaque switching and *wor1*Δ cells can not switch to or maintain the opaque phenotype [12]–[14]. To elucidate the relationship between *CZF1* and *WOR1*, we investigated whether *WOR1* was also required for anaerobically induced white-opaque switching, whether *CZF1* required the presence of *WOR1* to induce switching and, vice versa, whether *WOR1* required an intact *CZF1* gene to induce switching. For this purpose, we constructed two independent *wor1*Δ mutants of strain WO-1 using the *SAT1*-flipping strategy and expressed the *CZF1* and *WOR1* genes from the Tet-inducible promoter in the wild type and in the *czf1*Δ and *wor1*Δ mutants. In line with previously reported results [12]–[14], no spontaneous switching to the opaque phase was observed in the *wor1*Δ mutants, and neither anaerobic conditions (see below) nor transient expression of *CZF1* or *WOR1* from the Tet-inducible promoter could induce white-opaque switching in the absence of the endogenous *WOR1* gene (Table 3). These results demonstrated that *CZF1*-mediated, anaerobically induced switching to the opaque phase requires the master regulator *WOR1*. In contrast, doxycycline-induced expression of *WOR1* efficiently induced white-opaque switching in *czf1*Δ mutants (Table 3). Similarly, a transient expression of *CZF1* from the Tet-inducible promoter was sufficient to induce white-opaque switching even in a *czf1*Δ background, indicating that in contrast to *WOR1* no further expression of *CZF1* is required to maintain the opaque phenotype once switching to the opaque phase has been induced. To investigate whether Czf1p induces *WOR1* expression, we compared the *WOR1* mRNA levels in strains expressing *CZF1* under control of the Tet-inducible promoter as well as in strains containing a control construct with *GFP* instead of *CZF1*. As can be seen in Fig. 3, *WOR1* was expressed at very low levels in the control strains and in the strains containing the P_tet_-*CZF1* fusion in the absence of doxycycline (between 0.03 and 0.05% of *ACT1* mRNA levels). However, doxycycline-induced *CZF1* expression resulted in high *WOR1* expression levels (13–14% of *ACT1* mRNA levels, i.e., a more than 300-fold induction). Altogether, these results indicated that the role of *CZF1* in the environmentally induced white-opaque switching is the activation of *WOR1* expression in response to anaerobic conditions, which then allows switching to and maintenance of the opaque phase.

**Table 3:** Frequency of white-opaque switching in strains expressing *CZF1* or *WOR1* in wild-type, *czf1*Δ, and *wor1*Δ backgrounds.

Strain	Relevant genotype	no. of white, opaque, and mixed white/opaque (w/o) colonies^a^					
		+Dox			−Dox		
		white	opaque	w/o	white	opaque	w/o
WTET1-CZF1A	P_tet_-*CZF1*	39	612	25	779	1	10
WTET1-CZF1B	P_tet_-*CZF1*	27	538	11	681	1	5
WTET6-WOR1A	P_tet_-*WOR1*	110	150	13	567	0	19
WTET6-WOR1B	P_tet_-*WOR1*	198	92	13	559	0	18
WCZF1M4A	*czf1*Δ/*czf1*Δ	328	0	0	454	0	0
WCZF1M4B	*czf1*Δ/*czf1*Δ	399	0	0	542	0	0
WCZF1M4TET1-CZF1A	*czf1*Δ/*czf1*Δ	3	360	0	450	0	0
	P_tet_-*CZF1*						
WCZF1M4TET1-CZF1B	*czf1*Δ/*czf1*Δ	15	351	3	347	0	0
	P_tet_-*CZF1*						
WCZF1M4TET6-WOR1A	*czf1*Δ/*czf1*Δ	4	118	0	423	0	0
	P_tet_-*WOR1*						
WCZF1M4TET6-WOR1B	*czf1*Δ/*czf1*Δ	6	156	1	356	0	0
	P_tet_-*WOR1*						
WWOR1M6A	*wor1*Δ/*wor1*Δ	378	0	0	410	0	0
WWOR1M6B	*wor1*Δ/*wor1*Δ	406	0	0	563	0	0
WWOR1M6TET1-CZF1A	*wor1*Δ/wor*1*Δ	370	0	0	275	0	0
	P_tet_-*CZF1*						
WWOR1M6TET1-CZF1B	*wor1*Δ/wor*1*Δ	351	0	0	159	0	0
	P_tet_-*CZF1*						
WWOR1M6TET6-WOR1A	*wor1*Δ/wor*1*Δ	204	0	0	329	0	0
	P_tet_-*WOR1*						
WWOR1M6TET6-WOR1B	*wor1*Δ/wor*1*Δ	237	0	0	163	0	0
	P_tet_-*WOR1*						

a Results are from two or three experiments performed in parallel for each strain.

### Genomic alterations place white-opaque switching under environmental control

**Figure 4:**
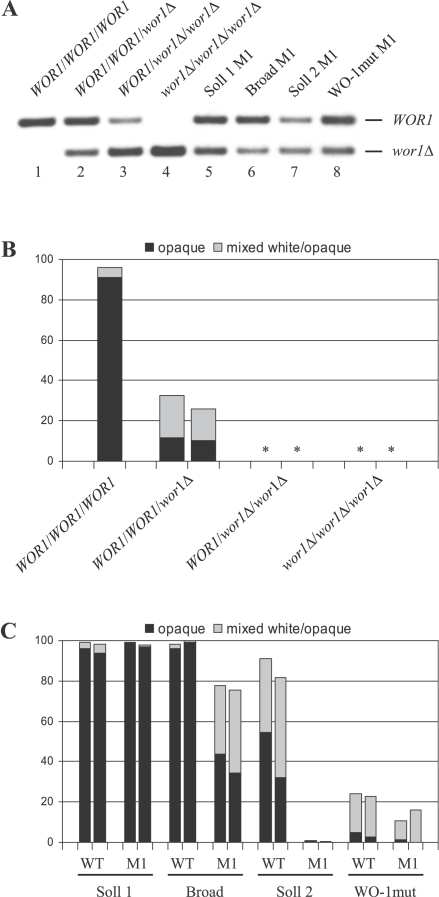
WOR1 copy number and inducibility of white-opaque switching in different stocks of strain WO-1 and mutant derivatives. (A) Southern hybridization of *Eco*RI/*Xho*I-digested genomic DNA of various strains with a probe from the *WOR1* upstream region. Lanes 1–4 show the hybridization pattern of the wild-type strain WO-1 (*WOR1*/*WOR1*/*WOR1*) and mutants in which one (*WOR1*/*WOR1*/*wor1*Δ), two (*WOR1*/*wor1*Δ/*wor1*Δ), or all three *WOR1* alleles (*wor1*Δ/*wor1*Δ/*wor1*Δ) were deleted. Lanes 5–8 show the hybridization pattern of mutants of additional WO-1 stocks in which one *WOR1* allele was replaced by the *SAT1* flipper cassette. The identity of the hybridizing DNA fragments is indicated. The size of the fragment representing the *wor1*Δ allele remains the same after excision of the *SAT1* flipper cassette due to an introduced *Xho*I site. (B) White-phase cells of the wild-type strain WO-1 and two independently constructed series of mutants lacking one, two, or all three *WOR1* alleles were incubated for two days under anaerobic conditions at 25°C on Lee's agar plates and subsequently grown for one week under aerobic conditions to determine the percentage of opaque (black bars) and mixed white/opaque colonies (grey bars). Stars (*) indicate that no opaque colonies were observed in these strains. (C) White-phase cells of different WO-1 stocks and mutant derivatives were incubated for two days under anaerobic conditions at 37°C on Lee's agar plates and the percentage of opaque and mixed white/opaque colonies was determined as described in (B). Results are from two experiments performed in parallel for each strain. WT: wild-type parent, M1: mutant in which one *WOR1* allele was deleted.

Since all experiments described above had been performed with the *MTL*α strain WO-1, we investigated if white-opaque switching would also be induced under anaerobic conditions in *MTL* **a** and in other *MTL*α strains. Therefore, we tested a set of three *MTL* **a** (12C, L26, P37005) and three *MTL*α strains (19F, P57072, P78048) provided by the Soll laboratory. However, none of these strains could be induced to switch from the white to the opaque phase under anaerobic conditions or in the presence of ketokonazole (Table 4 and data not shown), indicating that strain WO-1 exhibits unique characteristics that allow white-opaque switching to become inducible by environmental conditions. During the construction of the *wor1*Δ mutants we noted that three rounds of allelic replacement were required to generate null mutants (Fig. 4A, lanes 1–4), suggesting that the possession of an additional copy of the master regulator *WOR1*, whose expression levels determine whether cells adopt the white or the opaque phase [12]–[14], might have placed white-opaque switching under environmental control. Indeed, deletion of one *WOR1* allele decreased white-opaque switching under anaerobic conditions, although the mutants containing two instead of three *WOR1* alleles could still be induced to switch to the opaque phase (Fig. 4B). Deletion of two of the three alleles abolished the inducibility of white-opaque switching.

**Table 4:** Anaerobically induced white-opaque switching in *MTL* **a** and *MTL*α strains and derivatives carrying an additional *WOR1* copy.

Strain	no. of white, opaque, and mixed white/opaque (w/o) colonies^a^					
	anaerobic			aerobic		
	white	opaque	w/o	white	opaque	w/o
WO-1	2	84	0	91	0	0
19F	141	0	0	92	0	0
19F-WOR1	135	0	0	171	0	0
L26	126	1	5	169	0	0
L26-WOR1A	69	11	35	155	0	0
L26-WOR1B	34	8	28	75	0	0
P37005	120	0	0	130	0	0
P37005-WOR1A	151	3	6	156	1	0
P37005-WOR1B	132	1	1	128	0	0
P78048	138	0	0	101	0	0
P78048-WOR1A	102	2	2	103	0	0
P78048-WOR1B	165	0	1	130	0	0

a Results from two independently tested colonies were combined in each experiment.

To investigate whether the *WOR1* duplication was a peculiarity of the WO-1 stock maintained in our laboratory, we obtained strain WO-1 from the Soll lab. When this strain was transformed with the *WOR1* deletion cassette, Southern hybridization analysis of the resulting transformants showed that the strain also contained three *WOR1* alleles, as judged from the relative signal intensities of the bands representing intact *WOR1* and mutated *wor1*Δ alleles (Fig. 4A, lane 5). Comparative genome hybridization (CGH) experiments performed in the Berman lab demonstrated that the WO-1 stock that we obtained from the Soll lab (here referred to as Soll 1) as well as a WO-1 stock that was sequenced by the Broad institute (WO-1 Broad) were trisomic for chromosome 1 on which *WOR1* is located (A. Forche and J. Berman, personal communication). In contrast, another WO-1 stock obtained by the Berman lab from the Soll lab (Soll 2) contained only two chromosome 1 copies. Southern hybridization analysis of transformants of strains WO-1 Broad and WO-1 Soll 2 in which one *WOR1* allele was replaced by our deletion cassette confirmed that they contained three and two *WOR1* copies, respectively (Fig. 4A, lanes 6 and 7).

To investigate whether the acquisition of an additional *WOR1* copy was sufficient to place white-opaque switching under environmental control, we tested the anaerobic induction of switching in the other WO-1 stocks and their mutant derivatives in which one *WOR1* allele was deleted. As shown in Fig. 4C, switching was efficiently induced in strains WO-1 Soll 1 and WO-1 Broad. Interestingly, deletion of one of the three *WOR1* alleles had no effect on the inducibility of switching in WO-1 Soll 1 and only slightly reduced switching in WO-1 Broad. Switching was also efficiently induced in WO-1 Soll 2, which contains only two *WOR1* copies, although not to the same levels as in the other strains. However, deletion of one *WOR1* allele in this strain abolished the induction of switching by anaerobic conditions. These results demonstrated that the copy number of the master regulator *WOR1* affects the inducibility of white-opaque switching in a strain-dependent fashion, but trisomy for chromosome 1 and *WOR1* does not explain the environmental induction of switching in strain WO-1. This conclusion was further corroborated when we isolated a derivative of strain WO-1 Soll 1 in which the anaerobic induction of white-opaque switching was strongly reduced after prolonged passaging on agar plates (strain WO-1mut). We suspected that this strain might have lost one *WOR1* copy. However, Southern hybridization analysis of a transformant in which one *WOR1* allele was replaced by our deletion cassette demonstrated that the strain still retained three *WOR1* copies (Fig. 4A, lane 8) and the deletion of one copy did not significantly affect the anaerobically induced switching frequency of this mutant (Fig. 4C).

To study the effect of increasing the *WOR1* copy number on switching in other *MTL* homozygous *C. albicans* strains, we integrated an additional *WOR1* allele under control of its own regulatory sequences at an ectopic site into the genome of several of the non-inducible *MTL* **a** and *MTL*α strains mentioned above (see Materials and Methods). The results shown in Table 4 demonstrate that the effect of the additional *WOR1* copy on white-opaque switching depended on the strain background. Most strains still remained uninducible, while in two independent transformants of one strain (L26) the switching frequency under anaerobic conditions was significantly increased, albeit not to the levels observed in strain WO-1. Altogether, these results demonstrate that unstable genomic alterations that have occurred in strain WO-1 allow white-opaque switching to be inducible by environmental conditions.

### White-opaque switching is induced in the mammalian gastrointestinal tract

**Figure 5:**
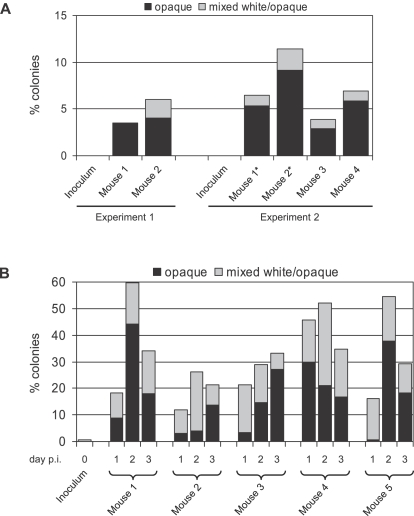
White-opaque switching is induced in the mammalian gastrointestinal tract. (A) Mice were inoculated intragastrically with 10^6^ white-phase cells of strain WO-1. Cells from the inoculum and cells recovered from the feces of the animals after 24 h (experiment 1 with mouse 1 and mouse 2) or 20 h (experiment 2 with mice 1 to 4) were grown on Lee's agar plates to determine the percentage of opaque (black bars) and mixed white/opaque colonies (grey bars). The stars indicate that mice 1 and 2 were infected a second time. In both experiments, no opaque colonies were present on the plates containing cells from the inoculum (243 and 194 white colonies). (B) Five antibiotic treated mice were inoculated intragastrically with 5×10^7^ white-phase cells of strain WO-1. Cells were recovered from the feces of the animals at one, two, or three days post infection (p.i.). The cells were grown on Lee's agar plates to determine the percentage of opaque (black bars) and mixed white/opaque colonies (grey bars). No opaque colonies were present on plates containing cells from the inoculum (436 white and 2 mixed white/opaque colonies).

Our finding that white-opaque switching can be induced in certain *C. albicans* strains under anaerobic conditions even at 37°C suggested that appropriate host niches in which such conditions are encountered, like the mammalian intestine, might promote white-opaque switching. To address this hypothesis, we infected mice intragastrically with white cells of strain WO-1 and collected the cells during the following days from the feces of the animals, i.e., after passage through the intestine. As can be seen in Fig. 5A, in a first set of experiments between 4 and 10% of the cells recovered after one day from the feces had switched to the opaque phase, i.e., the frequency of switching was increased by up to two orders of magnitude as compared with the spontaneous switching rate. No cells were recovered from the feces after 48 h and on the following days, indicating that the mice had efficiently eliminated the *C. albicans* cells from the intestine. In order to achieve a prolonged colonization of the intestine, we performed a second set of experiments using antibiotic-treated mice and a higher inoculum of *C. albicans* cells. As can be seen in Fig. 5B, white-opaque switching was further induced under these conditions, with the percentage of opaque and sectored colonies reaching more than 40% in some animals during the 3 days on which the cells were recovered from the feces. These results demonstrated that the conditions encountered in the mammalian intestine induced white-opaque switching in a large proportion of the cell population. We also tested two of the strains that did not switch to the opaque phase in response to anaerobic conditions, the *MTL*α strain 19F and the *MTL* **a** strain L26, under the same *in vivo* conditions. However, no significant induction of white-opaque switching was observed in 10 antibiotic-treated mice (5 infected mice for each of the two strains), indicating that the ability to switch to the opaque phase in the mammalian gastrointestinal tract is linked to the ability of *C. albicans* to switch in response to anaerobic conditions.

## Discussion

Since the discovery of white-opaque switching in *C. albicans* more than 20 years ago [4], it has been known that environmental conditions can influence the frequency of switching between the two phases. Opaque cells are stable only at lower temperatures and revert to the white phase at 37°C, and the switching frequency from the white to the opaque phase can also be modulated by the growth conditions [10]. Nevertheless, white-opaque switching was thought to be a stochastic process that occurs spontaneously at a relatively low frequency, which allows the two phases to be stably maintained and yet ensures that a population also contains cells of the opposite phase that may be better adapted to new host niches encountered during an infection [10].

Our observation that anaerobic conditions induce white cells to switch *en masse* to the opaque phase came as a surprise for several reasons. In a mammalian host anaerobic niches should be encountered within the body, i.e., at 37°C, a temperature at which opaque cells are unstable. In contrast, it has been suggested that a preferred host niche for opaque cells is skin, where the temperature is lower and which should represent a largely aerobic environment [9],[11]. In addition, a comparison of the gene expression patterns of white and opaque cells indicated that opaque cells exhibit an oxidative metabolism whereas white cells express a fermentative metabolism, pointing to a metabolic specialization of the two phases to the nutrients available in different anatomical sites [28]. Anaerobic conditions would not favor an oxidative metabolism and were therefore not expected to represent a suitable environment for opaque cells. However, our findings are in line with a recent report that anaerobic conditions stabilize the opaque phase at 37°C and that opaque cells can even mate at 37°C under anaerobic conditions [29].

Only a minority of *C. albicans* strains can switch to the opaque phase, as most strains are heterozygous at the mating type locus and first have to become *MTL* homozygous to relieve the inhibition of white-opaque switching by the **a**1–α2 repressor. Interestingly, deletion of one allele of the hemoglobin response gene *HBR1* in a heterozygous *MTL* **a**/α strain has been found to result in repression of *MTL*α1 and *MTL*α2 expression and allowed white-opaque switching to occur with the same low frequency as in *MTL* homozygous strains [30]. Chromosomal alterations are therefore a prerequisite for cells to become switching-competent, either by mitotic recombination, chromosome loss and duplication of the homologous chromosome, or loss of one allele of a regulatory gene [5],[7],[30],[31]. It is intriguing that additional genomic alterations allow white-opaque switching to become inducible by environmental conditions. We found that different stocks of strain WO-1 contain three *WOR1* copies due to chromosome 1 trisomy. However, although the *WOR1* copy number affected the inducibility of switching, other genomic alterations that are known to have occurred in strain WO-1 [32] seem to be responsible for the anaerobic induction, as white-opaque switching could also be induced in a strain that had only two copies of chromosome 1 and *WOR1*. Of note, the induction of white-opaque switching and the effect of deleting one *WOR1* allele differed between various stocks of strain WO-1, and CGH analysis showed that these strains differ from one another by additional genomic alterations (A. Forche and J. Berman, personal communication). Trisomy for chromosome 1 has also been described in certain stocks of the *MTL* **a**/α strain CAI4, a derivative of the model strain SC5314 [33], and other chromosomal alterations including loss and duplications as well as translocations involving different chromosomes are quite common in *C. albicans* [34]. Such genomic alterations often confer new phenotypes and have been suggested to be used as a mechanism of adaptation to new environments in this fungal pathogen [35]–[39]. The inducibility of white-opaque switching in an *MTL* homozygous strain is a novel phenotype gained by such genomic alterations.

The *WOR1* regulatory region is very large and it is likely to be subject to multiple regulatory inputs [12]. In addition to the Czf1p-mediated anaerobic induction of white-opaque switching, it is therefore possible that additional environmental signals also induce *WOR1* expression and switching of white cells to the opaque phase, possibly after other genomic alterations have occurred. Genomic alterations may alter the balance between the levels of environmentally sensitive positive and negative regulators of white-opaque switching, explaining the differences in the efficiency of switching induction and its dependence on *WOR1* copy number in various derivatives of strain WO-1.

The findings described in this study also shed new light on our view of the *C. albicans* life cycle. As stated above, only a minority of *C. albicans* strains are *MTL* homozygous and can switch to the opaque phase. As white-opaque switching itself occurs only at a relatively low frequency, opaque cells of opposite mating type from different strains would be expected to encounter one another very rarely in nature to allow mating and genetic exchange, in line with the observation that *C. albicans* populations are largely clonal [40]. For rare opaque cells in mixed *MTL* **a** and *MTL*α populations to come into contact, the surrounding white cells form a biofilm that is induced by the pheromone secreted from the opaque cells and allows stabilization of the pheromone gradient along which the shmoos of the opaque cells grow towards each other [41]. Our observation that specific environmental conditions can induce mass switching of white cells to the opaque phase in some strains suggests that in appropriate host niches the formation of such biofilms may not be required to allow mating, as a frequent contact of opaque cells of the two populations would be ensured. Such strains seem to be rare, and it will be important to identify an *MTL* **a** strain that, like the *MTL*α strain WO-1, switches to the opaque phase in response to anaerobic conditions or other signals encountered in the host to test this hypothesis. If more such inducible strains exist, they might represent a subpopulation of *C. albicans* in which genetic exchange occurs more frequently.

Growth on sorbose selects for strains that can utilize sorbose, which occurs after loss of one chromosome 5 homolog, and then results in strains that are *MTL* homozygous and switching-competent [5],[37]. One might speculate that certain environmental conditions could select for or even induce the chromosomal alterations that are necessary for white-opaque switching to become inducible and thus allow efficient mating when two strains occupy such a niche. The mammalian gastrointestinal tract, in which mating has been shown to be highly facilitated [29] and which we have shown here to induce white-opaque switching, might represent such a niche, as it is the normal habitat of *C. albicans* in its commensal state.

## Materials and Methods

### Strains and growth conditions

*C. albicans* strains used in this study are listed in Table 1. All strains were stored as frozen stocks with 15% glycerol at −80°C. The strains were subcultured separately in the white and opaque phases at room temperature on agar plates containing Lee's medium, pH 6.8 [42], and 5 µg ml^−1^ phloxine B, which selectively stains opaque colonies pink [43].

Incubation under anaerobic conditions was performed by plating appropriate dilutions of white-phase overnight cultures on Lee's agar plates and incubating the plates for different periods of time at 25°C or 37°C in an anaerobic jar (Anaerocult, Merck KGaA, Darmstadt, Germany) that generates an oxygen-free milieu in a CO_2_ atmosphere (18% CO_2_) within one hour. The plates were then transferred to aerobic conditions and further incubated at 25°C or at room temperature for 5–7 days to allow the development of visible colonies. Alternatively, cells were recovered from the plates after the anaerobic incubation period and used for microscopy or plated on fresh agar plates that were incubated under aerobic conditions for colony formation. For growth under anaerobic conditions in liquid medium, a 50 ml plastic tube with Lee's medium and the oxygen indicator resazurine was inoculated with white-phase cells of strain WO-1, flushed for 10 min with nitrogen gas to remove oxygen, sealed, and incubated for 2 days at 37°C. The cells were then appropriately diluted, spread on Lee's agar plates, and incubated for one week at room temperature to determine the percentage of white and opaque colonies. Treatment with ergosterol biosynthesis inhibitors was performed by diluting an overnight culture of white-phase cells 10^−2^ into Lee's medium containing the indicated concentrations of lovastatin or ketoconazole (stock solutions were 10 mg ml^−1^ lovastatin in 15% [vol/vol] ethanol, 0.25% [wt/vol] NaOH; 2 mg ml^−1^ ketoconazole in DMSO) or the corresponding solvent without drug. The cultures were grown for two days at 30°C, plated at an appropriate density on Lee's agar plates and incubated for one week at 25°C to allow colony formation. To test for a synergistic effect of ketoconazole and anaerobic conditions, white cells of strain WO-1 grown overnight in Lee's medium were spread on Lee's agar plates containing 5 µg ml^−1^ ketoconazole or DMSO only and incubated at 25°C in an anaerobic jar. The cells were recovered from the plates after 24 h and 48 h, spread at an appropriate density on Lee's agar plates, and incubated for one week at room temperature under aerobic conditions to allow colony formation. For determining the spontaneous switching frequency of the strains, several thousand colonies (100–300 colonies per plate) were counted. To test the effect of gene deletions on the inducibility of white-opaque switching of strain WO-1, the effect of ergosterol biosynthesis inhibitors on switching, and the induction of switching by anaerobic conditions in other strains, only a few hundred colonies were usually counted. In all experiments several independent white colonies of each strain were tested.

### Plasmid constructions

To express *CZF1*, *HAP41*, *HAP42*, *HAP43*, *RFG1*, *UPC2*, and *WOR1* from the Tet-inducible promoter [24], the coding sequences of these genes were amplified by polymerase chain reaction (PCR) with the primer pairs CZF1-1/CZF1-2, HAP411/HAP412, HAP421/HAP422, HAP431/HAP432, RFG1/RFG3, UPC2-1/UPC2-2, and WOR1-1/WOR1-2, respectively (primer sequences are given in Table S1). The PCR products were digested at the introduced *Sal*I or *Xho*I and *Bgl*II sites and cloned into the *Sal*I/*Bgl*II-digested pNIM1 [24] or a derivative, pNIM6, in which the *TEF3* transcription termination sequence was substituted for the *ACT1* transcription termination sequence, resulting in plasmids pTET1-CZF1, pTET1-HAP41, pTET6-HAP42, pTET1-HAP43, pTET6-RFG1, pTET6-UPC2, and pTET6-WOR1. To generate the *CZF1* deletion construct, the *CZF1* upstream and downstream regions were amplified with the primer pairs CZF1-3/CZF1-4 and CZF1-5/CZF1-6, respectively, digested at the introduced *Apa*I/*Xho*I and *Sac*II/*Sac*I sites, and cloned on both sides of the *SAT1* flipper cassette in plasmid pSFS2 [27] to generate pCZF1M2. To obtain a *WOR1* deletion construct, the *WOR1* upstream and downstream regions were amplified with the primer pairs WOR1-3/WOR1-4 and WOR1-5/WOR1-6, respectively, digested at the introduced *Apa*I/*Xho*I and *Sac*II/*Sac*I sites, and substituted for the *CZF1* flanking sequences in pCZF1M2 to generate pWOR1M2. To introduce an additional *WOR1* copy into the *ACT1* locus of *MTL* **a** and *MTL*α strains, a *Kpn*I-*Cla*I fragment from pTET6-WOR1 containing the *WOR1* coding sequence, the *TEF3* transcription termination sequence, and part of the *caSAT1* selection marker was first cloned into the *Kpn*I/*Cla*I-digested pMPG2S [44] to result in pWOR1-1. A part of the *WOR1* coding region and upstream sequences were then amplified with the primers WOR1-9 and WOR1-10 and the PCR product digested with *Nco*I/*Eco*RI and cloned between the same sites in pWOR1-1 to generate pWOR1-2. Additional *WOR1* upstream sequences were amplified with the primers WOR1-11 and WOR1-12, digested at the introduced *Sph*I site and at a naturally occurring *Nco*I site, and cloned together with an *ACT1* fragment that was amplified with the primers ACT39 and ACT40 and digested with *Apa*I/*Sph*I into the *Apa*I/*Nco*I-digested pWOR1-2. The resulting plasmid pWOR1-3 contains 9 kb of *WOR1* upstream sequences and the *WOR1* coding region fused to the *TEF3* transcription termination sequence, the *caSAT1* selection marker, and flanking *ACT1* sequences for genomic integration.

### *C. albicans* transformation

*C. albicans* strains were transformed by electroporation [45] with gel-purified inserts from the plasmids described above. Nourseothricin-resistant transformants were selected on YPD agar plates containing 200 µg ml^−1^ nourseothricin (Werner Bioagents, Jena, Germany) as described previously [27]. Single-copy integration of all constructs was confirmed by Southern hybridization.

### Southern hybridization

Genomic DNA from *C. albicans* strains was isolated as described previously [46]. 10 µg of DNA was digested with appropriate restriction enzymes, separated on a 1% agarose gel and, after ethidium bromide staining, transferred by vacuum blotting onto a nylon membrane and fixed by UV crosslinking. Southern hybridization with enhanced chemiluminescence-labeled probes was performed with the Amersham ECL™ Direct Nucleic Acid Labelling and Detection System (GE Healthcare, Braunschweig, Germany) according to the instructions of the manufacturer.

### Fluorescence microscopy

Cells from white and opaque colonies of the strains carrying *GFP* reporter fusions were grown for 18 h at 25°C in liquid Lee's medium and aliquots were spotted on microscope slides. Fluorescence microscopy was performed with a Zeiss Axioplan microscope equipped for epifluorescence microscopy with a 50 W mercury high pressure bulb and a Zeiss fluorescein-specific filter.

### RNA isolation and real-time RT-PCR

Overnight cultures of strains WTET1-CZF1A and -B and WNIM1A and -B were diluted 10^−2^ in liquid Lee's medium with or without 50 µg ml^−1^ doxycycline and grown for 18 h at 30°C. Total RNA was isolated using the RNeasy Mini Kit (Qiagen, Hilden, Germany) and treated with Turbo DNA-free DNase (Ambion, Austin, TX). Total RNA from each sample was linearly reverse-transcribed using Superscript III Super Mix (Invitrogen, Karlsruhe, Germany), and cDNA was amplified by quantitative PCR with the primers WOR1LRT and WOR1RRT (for *WOR1*) and ACT1RT and ACT2RT (for *ACT1*), as monitored by Sybr Green fluorescence in a MyiQ real-time PCR detection system (Bio-Rad Labratories, Hercules, CA). The signals for *WOR1* were normalized to the *ACT1* transcript level for each strain and culture condition.

### *In vivo* experiments

White-phase cells of strain WO-1 were grown overnight in Lee's medium, washed two times in phosphate-buffered saline (PBS), and adjusted to a density of 10^7^ cells per ml. In a first experiment, two male Balb/c mice were intragastrically inoculated with 10^6^ cells. After 24 h and on the following days, the feces of the mice were collected, homogenized in sterile water, and a dilution series was spread on Lee's agar plates containing 10 µg ml^−1^ gentamycin and incubated for one week at room temperature to determine the percentage of white and opaque colonies. The experiment was repeated one week later with the same two mice and two additional mice. A third experiment was performed with antibiotic-treated mice. For this purpose, five Balb/c mice were fed with 1 mg ml^−1^ tetracycline, 2 mg ml^−1^ streptomycin, and 0.1 mg ml^−1^ gentamycin in their drinking water, starting from day 4 prior to infection. The mice were infected as described above with 5×10^7^ white-phase cells of strain WO-1 and the fungal cells were recovered from the feces of the animals on the following three days and plated on Lee's agar plates containing 50 µg ml^−1^ chloramphenicol to determine the percentage of white and opaque colonies. The latter experiment was repeated in an identical fashion with strains 19F and L26.

### Sequence data

Sequence data for *Candida albicans* was obtained from the *Candida* Genome Database (http://www.candidagenome.org/).

## Supporting Information

Table S1 Primers Used in This Study.
(0.06 MB DOC)

## References

[1] Whiteway M, Bachewich C (2007). Morphogenesis in *Candida albicans*.. Annu Rev Microbiol.

[2] Brown AJ, Gow NA (1999). Regulatory networks controlling *Candida albicans* morphogenesis.. Trends Microbiol.

[3] Whiteway M, Oberholzer U (2004). *Candida* morphogenesis and host-pathogen interactions.. Curr Opin Microbiol.

[4] Slutsky B, Staebell M, Anderson J, Risen L, Pfaller M (1987). “White-opaque transition”: a second high-frequency switching system in *Candida albicans*.. J Bacteriol.

[5] Miller MG, Johnson AD (2002). White-opaque switching in *Candida albicans* is controlled by mating-type locus homeodomain proteins and allows efficient mating.. Cell.

[6] Lockhart SR, Pujol C, Daniels KJ, Miller MG, Johnson AD (2002). In *Candida albicans*, white-opaque switchers are homozygous for mating type.. Genetics.

[7] Wu W, Lockhart SR, Pujol C, Srikantha T, Soll DR (2007). Heterozygosity of genes on the sex chromosome regulates *Candida albicans* virulence.. Mol Microbiol.

[8] Kvaal CA, Srikantha T, Soll DR (1997). Misexpression of the white-phase-specific gene *WH11* in the opaque phase of *Candida albicans* affects switching and virulence.. Infect Immun.

[9] Kvaal C, Lachke SA, Srikantha T, Daniels K, McCoy J (1999). Misexpression of the opaque-phase-specific gene *PEP1* (*SAP1*) in the white phase of *Candida albicans* confers increased virulence in a mouse model of cutaneous infection.. Infect Immun.

[10] Soll DR (1992). High-frequency switching in *Candida albicans*.. Clin Microbiol Rev.

[11] Lachke SA, Lockhart SR, Daniels KJ, Soll DR (2003). Skin facilitates *Candida albicans* mating.. Infect Immun.

[12] Zordan RE, Galgoczy DJ, Johnson AD (2006). Epigenetic properties of white-opaque switching in *Candida albicans* are based on a self-sustaining transcriptional feedback loop.. Proc Natl Acad Sci U S A.

[13] Huang G, Wang H, Chou S, Nie X, Chen J (2006). Bistable expression of *WOR1*, a master regulator of white-opaque switching in *Candida albicans*.. Proc Natl Acad Sci U S A.

[14] Srikantha T, Borneman AR, Daniels KJ, Pujol C, Wu W (2006). *TOS9* regulates white-opaque switching in *Candida albicans*.. Eukaryot Cell.

[15] Hughes AL, Todd BL, Espenshade PJ (2005). SREBP pathway responds to sterols and functions as an oxygen sensor in fission yeast.. Cell.

[16] Davies BS, Rine J (2006). A role for sterol levels in oxygen sensing in *Saccharomyces cerevisiae*.. Genetics.

[17] Silver PM, Oliver BG, White TC (2004). Role of *Candida albicans* transcription factor Upc2p in drug resistance and sterol metabolism.. Eukaryot Cell.

[18] MacPherson S, Akache B, Weber S, De Deken X, Raymond M (2005). *Candida albicans* zinc cluster protein Upc2p confers resistance to antifungal drugs and is an activator of ergosterol biosynthetic genes.. Antimicrob Agents Chemother.

[19] Kwast KE, Burke PV, Poyton RO (1998). Oxygen sensing and the transcriptional regulation of oxygen-responsive genes in yeast.. J Exp Biol.

[20] Johnson DC, Cano KE, Kroger EC, McNabb DS (2005). Novel regulatory function for the CCAAT-binding factor in *Candida albicans*.. Eukaryot Cell.

[21] Kadosh D, Johnson AD (2001). Rfg1, a protein related to the *Saccharomyces cerevisiae* hypoxic regulator Rox1, controls filamentous growth and virulence in *Candida albicans*.. Mol Cell Biol.

[22] Khalaf RA, Zitomer RS (2001). The DNA binding protein Rfg1 is a repressor of filamentation in *Candida albicans*.. Genetics.

[23] Brown DH, Giusani AD, Chen X, Kumamoto CA (1999). Filamentous growth of *Candida albicans* in response to physical environmental cues and its regulation by the unique *CZF1* gene.. Mol Microbiol.

[24] Park Y-N, Morschhäuser J (2005). Tetracycline-inducible gene expression and gene deletion in *Candida albicans*.. Eukaryot Cell.

[25] Vinces MD, Kumamoto CA (2007). The morphogenetic regulator Czf1p is a DNA-binding protein that regulates white opaque switching in *Candida albicans*.. Microbiology.

[26] Zordan RE, Miller MG, Galgoczy DJ, Tuch BB, Johnson AD (2007). Interlocking Transcriptional Feedback Loops Control White-Opaque Switching in *Candida albicans*.. PLoS Biol.

[27] Reuß O, Vik Å, Kolter R, Morschhäuser J (2004). The *SAT1* flipper, an optimized tool for gene disruption in *Candida albicans*.. Gene.

[28] Lan CY, Newport G, Murillo LA, Jones T, Scherer S (2002). Metabolic specialization associated with phenotypic switching in *Candida albicans*.. Proc Natl Acad Sci U S A.

[29] Dumitru R, Navarathna DH, Semighini CP, Elowsky CG, Dumitru RV (2007). In vivo and in vitro anaerobic mating in *Candida albicans*.. Eukaryot Cell.

[30] Pendrak ML, Yan SS, Roberts DD (2004). Hemoglobin regulates expression of an activator of mating-type locus α genes in *Candida albicans*.. Eukaryot Cell.

[31] Wu W, Pujol C, Lockhart SR, Soll DR (2005). Chromosome loss followed by duplication is the major mechanism of spontaneous mating-type locus homozygosis in *Candida albicans*.. Genetics.

[32] Chu WS, Magee BB, Magee PT (1993). Construction of an *Sfi*I macrorestriction map of the *Candida albicans* genome.. J Bacteriol.

[33] Chen X, Magee BB, Dawson D, Magee PT, Kumamoto CA (2004). Chromosome 1 trisomy compromises the virulence of *Candida albicans*.. Mol Microbiol.

[34] Selmecki A, Bergmann S, Berman J (2005). Comparative genome hybridization reveals widespread aneuploidy in *Candida albicans* laboratory strains.. Mol Microbiol.

[35] Rustchenko EP, Howard DH, Sherman F (1994). Chromosomal alterations of *Candida albicans* are associated with the gain and loss of assimilating functions.. J Bacteriol.

[36] Rustchenko EP, Howard DH, Sherman F (1997). Variation in assimilating functions occurs in spontaneous *Candida albicans* mutants having chromosomal alterations.. Microbiology.

[37] Janbon G, Sherman F, Rustchenko E (1998). Monosomy of a specific chromosome determines L-sorbose utilization: a novel regulatory mechanism in *Candida albicans*.. Proc Natl Acad Sci U S A.

[38] Perepnikhatka V, Fischer FJ, Niimi M, Baker RA, Cannon RD (1999). Specific chromosome alterations in fluconazole-resistant mutants of *Candida albicans*.. J Bacteriol.

[39] Kabir MA, Ahmad A, Greenberg JR, Wang YK, Rustchenko E (2005). Loss and gain of chromosome 5 controls growth of *Candida albicans* on sorbose due to dispersed redundant negative regulators.. Proc Natl Acad Sci U S A.

[40] Gräser Y, Volovsek M, Arrington J, Schönian G, Presber W (1996). Molecular markers reveal that population structure of the human pathogen *Candida albicans* exhibits both clonality and recombination.. Proc Natl Acad Sci U S A.

[41] Daniels KJ, Srikantha T, Lockhart SR, Pujol C, Soll DR (2006). Opaque cells signal white cells to form biofilms in *Candida albicans*.. Embo J.

[42] Bedell GW, Soll DR (1979). Effects of low concentrations of zinc on the growth and dimorphism of *Candida albicans*: evidence for zinc-resistant and -sensitive pathways for mycelium formation.. Infect Immun.

[43] Soll DR, Morrow B, Srikantha T (1993). High-frequency phenotypic switching in *Candida albicans*.. Trends Genet.

[44] Hiller D, Stahl S, Morschhäuser J (2006). Multiple cis-acting sequences mediate upregulation of the *MDR1* efflux pump in a fluconazole-resistant clinical *Candida albicans* isolate.. Antimicrob Agents Chemother.

[45] Köhler GA, White TC, Agabian N (1997). Overexpression of a cloned IMP dehydrogenase gene of *Candida albicans* confers resistance to the specific inhibitor mycophenolic acid.. J Bacteriol.

[46] Millon L, Manteaux A, Reboux G, Drobacheff C, Monod M (1994). Fluconazole-resistant recurrent oral candidiasis in human immunodeficiency virus-positive patients: persistence of *Candida albicans* strains with the same genotype.. J Clin Microbiol.

[47] Lockhart SR, Reed BD, Pierson CL, Soll DR (1996). Most frequent scenario for recurrent *Candida* vaginitis is strain maintenance with “substrain shuffling”: demonstration by sequential DNA fingerprinting with probes Ca3, C1, and CARE2.. J Clin Microbiol.

[48] Pujol C, Pfaller M, Soll DR (2002). Ca3 fingerprinting of *Candida albicans* bloodstream isolates from the United States, Canada, South America, and Europe reveals a European clade.. J Clin Microbiol.

[49] Park YN, Strauß A, Morschhäuser J (2004). The white-phase-specific gene *WH11* is not required for white-opaque switching in *Candida albicans*.. Mol Genet Genomics.

[50] Strauß A, Michel S, Morschhäuser J (2001). Analysis of Phase-Specific Gene Expression at the Single-Cell Level in the White-Opaque Switching System of *Candida albicans*.. J Bacteriol.

