# Supplementary material for: Environmental Induction of White–Opaque Switching in Candida albicans
Source: PLoS Pathog. 2008 Jun 13;4(6):e1000089. doi: 10.1371/journal.ppat.1000089 (PMC2405950; doi:10.1371/journal.ppat.1000089)
Supplement: Table S1 — Primers Used in This Study. (0.06 MB DOC) [file ppat.1000089.s001.doc]

**Table S1: Primers used in this study**

| Primer | Sequence ^a^ |
| --- | --- |
| ACT39 | 5`-ccttgagcatgccaattgaacacgg-3` |
| ACT40 | 5`-atatgggcccgggttaccgtataattcttttctaac-3` |
| ACT1RT ^b^ | 5`-AGTGTGACATGGATGTTAGAAAAGAATTATACGG-3` |
| ACT2RT ^b^ | 5`-ACAGAGTATTTTCTTTCTGGTGGAGCA-3` |
| CZF1-1 | 5`-AAATGTCGACAATGAGTTCAATACCCAATAT-3` |
| CZF1-2 | 5`-CCAACACAGAGATCTTATTTACTTCTG-3` |
| CZF1-3 | 5`-aatagggcccacccaacagtgttaatcttg-3` |
| CZF1-4 | 5`-CCATCTCGAGGGTCATTCCAATTGATATTGGG-3` |
| CZF1-5 | 5`-TTTTCCGCGGTATTGTTGAATACAGAAG-3` |
| CZF1-6 | 5`-CTTTGGTTGTAGAGCTCAAATACCCTC-3` |
| HAP411 | 5`-ATATGTCGACACAATGTATACTAACATTATTCTAGCTGC-3` |
| HAP412 | 5`-CCGTTAATTTTTTAGATCTCCTTCATACATTCATC-3` |
| HAP421 | 5`-TAACGTCGACGGAATGTCTCTGATGATAAATAAAGAACC-3` |
| HAP422 | 5`-CCAAAATAGCAACCAGATCTTAAATCACTTTTC-3` |
| HAP431 | 5`-GAATTTCAATGTCGACAACATGCCCGCAAAAGGTCC-3` |
| HAP432 | 5`-CTTCATACTGTAAGATCTAACTAATTATATGC -3` |
| RFG1 | 5`-ATATGTCGACATTATGTCTACTGCTATCTACTATTCAACTC-3` |
| RFG3 | 5`-CTACCATTTCTTCTTGGAGATCTATCTATACCCCAGG-3` |
| UPC2-1 | 5`-ATATCTCGAGATGATGATGACAGTGAAACAAGAATC -3` |
| UPC2-2 | 5`-ATATAGATCTAAGTACCGGTGTAATACGACCCAGAAG -3` |
| WOR1-1 | 5`-ATATGTCGACAATGTCTAATTCAAGTATAGTCCCTAC -3` |
| WOR1-2 | 5`-ATATAGATCTATTTCATATTCATAAACCCATTATC -3` |
| WOR1-3 | 5`-CAAAGTTTGGAGGGCCCTAAACATTCATTAAC -3` |
| WOR1-4 | 5`-GACATTGCTCGAGATTGAATTGAATTATAC -3` |
| WOR1-5 | 5`-GGGTCGTATTACCGCGGTACTTAGTTG -3` |
| WOR1-6 | 5`-TTTTGAGCTCTCTAAGTAGTATTGATC -3` |
| WOR1-9 | 5`-ggattcacaaccatggttgg-3` |
| WOR1-10 | 5`-tcgagaattcttattaaggtgac-3` |
| WOR1-11 | 5`-atatgcatgcggtcacaattagtcgtctgc-3` |
| WOR1-12 | 5`-ccaaccatggttgtgaatcc-3` |
| WOR1LRT ^b^ | 5`-CCACCAGCAGTCAGTACCAA-3` |
| WOR1RRT ^b^ | 5`-TAGTCATTGGCATGGGTTCA-3` |

^a^ Restriction sites introduced into the primers are underlined.

^b^ Primers used for real-time RT-PCR
